# Serglycin Across the Disease Spectrum: A Multifunctional Proteoglycan in Inflammation and Cancer

**DOI:** 10.3390/cimb48050454

**Published:** 2026-04-28

**Authors:** Eleftherios N. Athanasopoulos, Vassiliki T. Labropoulou, Achilleas D. Theocharis

**Affiliations:** 1 Biochemistry, Biochemical Analysis and Matrix Pathobiology Research Group, Laboratory of Biochemistry, Department of Chemistry, University of Patras, 26504 Patras, Greece; lefterhsathanasopoulos@gmail.com; 2 Hematology Division, Department of Internal Medicine, University Hospital of Patras, University of Patras, 26504 Patras, Greece; vaslabrop@upatras.gr

**Keywords:** proteoglycans, serglycin, inflammation, extracellular matrix, cancer, tumor microenvironment

## Abstract

The inflammatory response possesses a central role in human pathophysiology, regulating the tissue microenvironment and cell signaling. Inflammation occurs either as a symptom of homeostasis disturbance or as a driver for determining cell fate. In this context, cells recruit secreted cytokines, chemokines and intracellular mediators, in cooperation with their surrounding cellular components, to integrate inflammatory stimuli. The extracellular matrix (ECM) acts as a scaffold for shaping tissue structure and simultaneously undergoes continuous remodeling to provide a dynamic network for intercellular communication. Serglycin (SRGN) is the only known intracellular and extracellular proteoglycan, implicated in the formation of secretory vesicles and ECM reorganization. The regulatory roles of SRGN in the bioavailability of secreted factors, as well as SRGN pleiotropic interactions within the ECM, as well as with cell surface receptors, have emerged to beessential for inflammatory diseases and tumor progression. Its overexpression and excessive secretion, alongside its contribution to cell signaling, highlight the potential diagnostic and therapeutic aspects of SRGN in human diseases.

## 1. Introduction

Cellular homeostasis and physiology are largely affected by the surrounding microenvironment and extracellular stimuli. The extracellular matrix (ECM) is a complex network of secreted and transmembrane biomolecules, establishing cell and tissue integrity and communication through diverse interactions [1,2]. To substantiate active responses to the dynamic pathophysiological signals that cells integrate, the ECM undergoes consistent remodeling catalyzed by extracellular proteases, mainly matrix metalloproteases (MMPs), and successive protein synthesis and secretion by stromal cells. Cell–ECM and cell–cell crosstalk are mediated by pleiotropic subpopulations of biomolecules, including growth factors (GFs) and their receptors, cytokines, glycosaminoglycans (GAGs) and proteoglycans (PGs), acting as signal transducers in an autocrine and paracrine fashion [3].

PGs are predominantly localized within the ECM or anchored on the cell membrane but are also found intracellularly. PGs’ protein core acts as a scaffold for the covalent binding of GAGs in diverse combinations through O-glycosylation. Sulfation of their decorated GAGs confers a robust negative charge, upon which PGs mainly exert their functions [4,5]. Serglycin (SRGN) is the only intracellular PG characterized so far, being also found secreted within the ECM. Its small protein core (158aa) includes eight Ser/Gly juxtaposed repeats that serve as anchors for heparin (Hep), chondroitin sulfate (CS) and, to a lesser extent, heparan sulfate (HS) GAGs [6]. CS glycosylation is essential for SRGN binding to CD44 receptor, while there is intriguing evidence suggesting that hyaluronidases (HYALs) HYAL1/4 can degrade CS GAGs, highlighting new aspects of secreted SRGN metabolism [7,8]. SRGN intracellular presence and the glycosylation pattern have been associated with the formation of secretory vesicles in hematopoietic cells and, markedly, in mast cells (MCs). It has become increasingly evident that SRGN overexpression and secretion are constitutive not only in inflammatory cells but also in malignancies [9].

**Figure 1:**
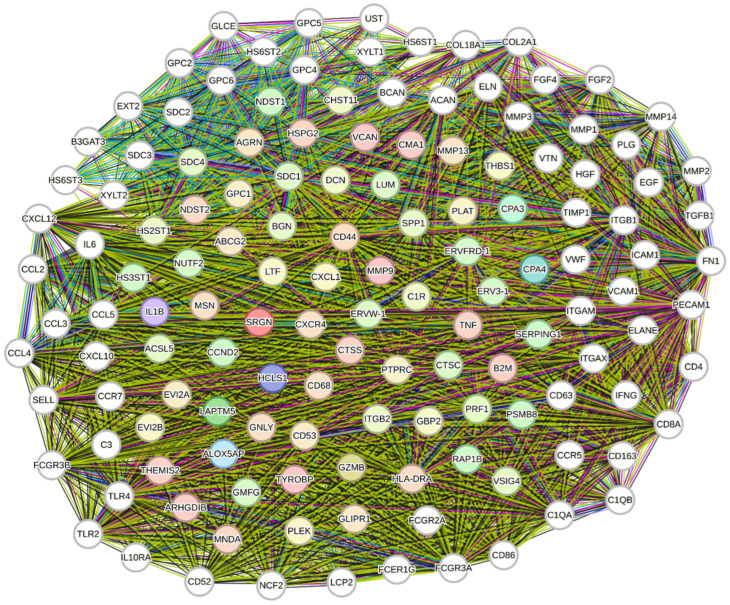
Network of predicted functional partners for human SRGN. Molecules depicted with color are directly connected to SRGN (1st shell), while white-colored molecules (2nd shell) have an indirect relationship with SRGN due to their interaction with 1st-shell nodes. The confidence score is >0.4 (http://string-db.org/).

**Figure 2:**
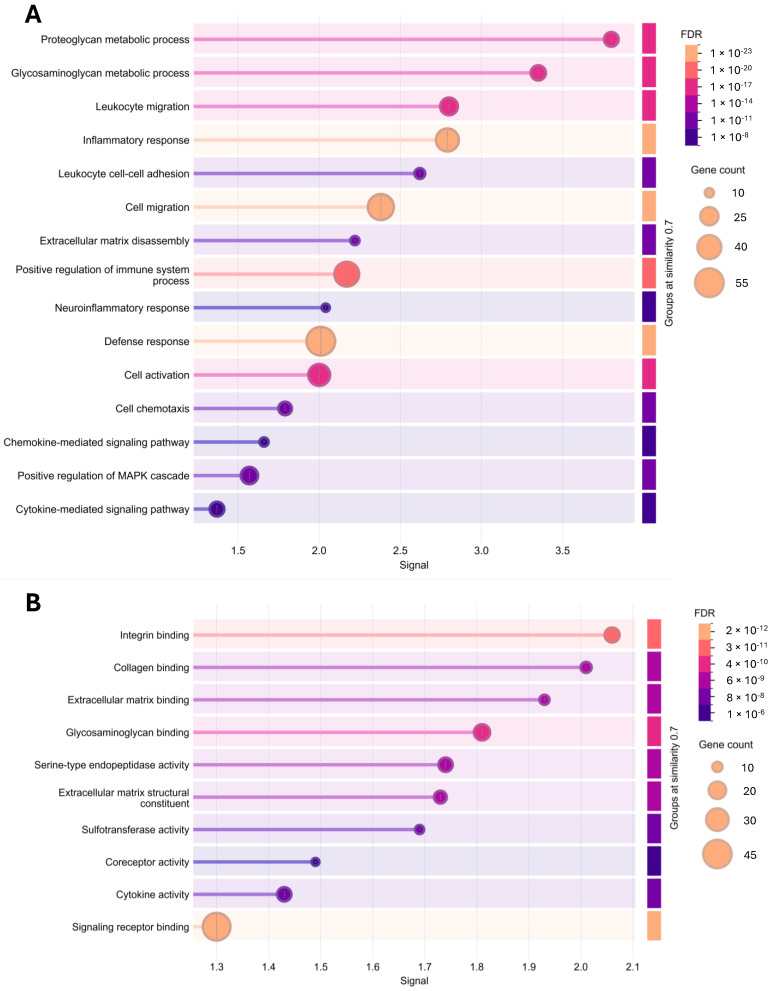
Functional enrichments in SRGN network revealed gene clusters (gene ontology dot plots) associated with annotated biological processes (**A**) and molecular functions (**B**) (http://string-db.org/). FDR: false discovery rate; this measure describes how significant the enrichment is. Shown are *p*-values corrected for multiple testing within each category using the Benjamini–Hochberg procedure. Signal: the signal is defined as a weighted harmonic mean between the observed/expected ratio and −log(FDR). Gene count: indicates how many proteins in the network are annotated with a particular term.

The predicted functional network of SRGN encompasses numerous proteins, including growth factors, cytokines, proteolytic enzymes, ECM molecules, cell surface receptors and signaling molecules, and intracellular proteins. Figure 1 presents molecules that are directly connected to SRGN, along with those that have an indirect relationship via interactions with molecules directly linked to SRGN (http://string-db.org/). Functional enrichments in the SRGN network have revealed gene clusters associated with several biological processes, including GAG/PG metabolism and ECM disassembly, cell activation and signaling, positive regulation of immune cells’ activation and response, and neuroinflammation (Figure 2A) (http://string-db.org/). Functional enrichments in the SRGN network also include genes implicated in several molecular functions, such as binding to cell surface receptors and activation, as well as binding to ECM molecules and matrix organization (Figure 2B) (http://string-db.org/).

High SRGN levels have been correlated with immunological responses and inflammatory phenotype both in vitro and in vivo, as well as in patient tissues regarding chronic, degenerative disease and cancer. It has been found to modulate the expression and potent signaling of interleukins (ILs) and chemokines, namely, IL-1β, tumor necrosis factor-α (TNF-α) and transforming growth factor-β (TGF-β), participating in osteoarthritis (OA) deterioration, neurodegeneration and cancer progression of multiple malignancies, acting as an upstream regulator of inflammatory cell signaling [9,10,11,12]. This article focuses on the diverse roles of SRGN in signal transduction and ECM reconstruction as described in the recent literature, aiming to decipher the molecular mechanisms and events that SRGN orchestrates in an inflammatory setting.

## 2. Roles of SRGN in Inflammation-Related Diseases

### 2.1. Immune Response

**Figure 3:**
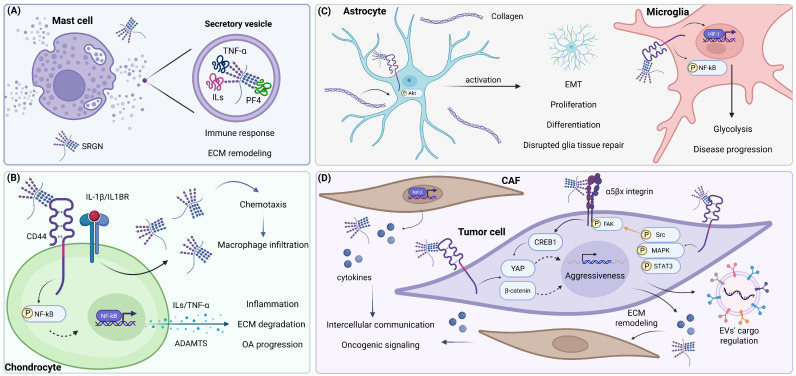
(**A**) SRGN dominates mast cells’ homeostasis. Mast cells express and secrete high levels of SRGN. Intracellularly, chemokines, GFs, proteases and other factors bind to SRGN GAGs within secretory vesicles. SRGN regulates their bioavailability and stimulates the proinflammatory response. (**B**) Continuous ECM degradation and reconstruction deteriorate OA in a SRGN-dependent manner. IL-1β induces SRGN expression and secretion, providing extracellular chemotactic signals and macrophage infiltration in the surrounding pathological joint. SRGN tethers to chondrocytes’ CD44 and promotes the NF-kB cascade, thus upregulating the extracellular function of ILs, chemokines and proteases, further promoting degenerative OA. (**C**) Glial cells incorporate SRGN/CD44 and collagen towards inflammation and disrupted glial niche repair. Astrocytic activation via SRGN/CD44 and SRGN/collagen interplay enhances astrocytes’ proliferation potential through Akt signaling, leading to a differentiated phenotype and impaired repair capacity in glial damage. Alongside, SRGN attenuates stroke-induced injury repair through the inflammatory SRGN/CD44/NF-kB axis and HIF-1-dependent metabolic reprogramming, redirecting microglia cells to glycolysis and disease progression. (**D**) SRGN provokes oncogenic signaling and intercellular crosstalk within the TME. SRGN/CD44 interaction amplifies the aggressive properties of tumor cells through Src, MAPK, STAT3, YAP and β-catenin pathways. Direct binding of SRGN to α3 integrin induces FAK activity, thereby activating CREB1 and concomitant YAP expression, while Src transactivates and enhances FAK activation. This signaling network orchestrates ECM remodeling, EMT and tumorigenic pro-inflammatory response. SRGN also mediates cancer–stroma cells’ communication through regulating EVs’ cargo and CAFs’ activation. CAFs experiencing hypoxia recruit antioxidant and autophagic mechanisms, implicating SRGN secretion and the formation of an inflammatory TME, concluding in tumor progression and malignancy. Created in BioRender. Athanasopoulos, E. (2026) https://BioRender.com/qyouzz7.

The importance of MCs in the innate immune system underscores the probable roles of SRGN in stimulating the inflammatory response. Lack of SRGN in mucosa MCs infected with *Salmonella typhimurium* has been shown to lead to failed granule organization, extensive bacterial colonization and intracellular accumulation, eventually resulting in an ineffective response [13]. It has also been documented that *Giardia intestinalis* intestinal infection provokes a SRGN-dependent secretion of IL-6 and expression of TNF-α and C-X-C motif chemokine ligand-2 (CXCL-2) in mice to counteract parasitic infection and weight loss [14]. SRGN also stores histamine in MCs’ vesicles through direct interaction of its GAG chains with different binding partners, therefore regulating their bioavailability and secretion [15]. pH alterations from granule acidic pH towards neutral pH after degranulation release histamine from Hep, allowing histamine to bind to its receptors and regulate cell proliferation, differentiation and wound healing (Figure 3A) [16]. Similarly, *Lacticaseibacillus casei JCM1134T* induces CCAAT/enhancer-binding protein α (C/EBPα) levels, thereby inhibiting SRGN expression via C/EBPα transcription factor (TF) activity. This results in insufficient granule formation and incompetent microbial elimination [17], while it is also well-documented that immature and permeable MC granules result in leakage of proteases within the cytosol and SRGN-dependent apoptosis [18,19].

SRGN is not limited to MC homeostasis but also participates in the manufacture of the bone marrow microenvironment. In this context, SRGN has been shown to permit the maturation of megakaryocytes (MKs) and platelet formation. Loss of SRGN in MKs is linked to excessive concentration of platelet factor 4 (PF4), TGF-β, IL-1β and TNF-α in the bone marrow and disrupted MKs’ differentiation in vitro and in vivo, accompanied by altered ECM composition with reduced laminin and increased collagen I levels. This phenomenon was GAG-dependent and mediated by defective retention and aberrant protein secretion in SRGN-knockout cells, rather than protein turnover, leading overall to a microenvironment inadequate for MK’s maturation. Apart from mild thrombocytopenia, SRGN^–/–^ mice also exhibited reduced numbers of hematopoietic stem cells and multipotent progenitors [20]. Platelet α-granules’ structure and quality were also altered upon SRGN depletion, exhibiting diminished fibrous and basic protein content [21]. An SRGN-enriched ECM of eosinophil cells is also crucial for the retention of chemokine ligand 26 (CCL26) via HS chains to provide chemotactic signals and eventually eosinophils’ migration, undergoing inflammatory activation [22].

### 2.2. Rheumatologic Disorders

CCL3 and chemotactic activity have also been linked to SRGN function towards inflammatory propagation in OA. Chondrocytes’ distortion within the synovial joints, followed by macrophage migration to the degraded OA ECM, occurs, in part, due to SRGN’s indirect immune response. In detail, SRGN expression was positively correlated with OA onset and macrophage accumulation in patients. Mechanistically, unrestrained IL-1β secretion from chondrocytes stimulated SRGN expression and secretion, and consequential SRGN-induced CCL3 expression and macrophage attraction, concluding in OA progression (Figure 3B) [23]. Different studies have proposed that IL-1β-dependent SRGN function in chondrocytes is initiated by SRGN tethering to CD44, provoking nuclear factor-kappa B (NF-kΒ) signaling and inflammation in cartilage degradation and arthritis (Figure 3B) [24]. In accordance with the above, late-stage nucleus pulposus cells display a fibrotic and inflammatory phenotype in intervertebral disc degeneration by activating NF-kB through extracellular SRGN, therefore inducing the expression and secretion of IL-1β, TNF-α and CCL3 for macrophages’ infiltration (Figure 3B) [25]. Correlational studies have also suggested that SRGN expression and serum SRGN are significantly upregulated together with (C-reactive protein) CRP in patients with idiopathic inflammatory myopathy, activating macrophage differentiation, cytokines’ secretion and disease progression [26]. Collaterally with SRGN/inflammation correlation, catabolic enzymes’ expression, such as A disintegrin and metalloproteinase with thrombospondin motifs 14 (ADAMTS14), in the synovial fluid and blood predict poor joint homeostasis, enhanced degradation, late OA stage and rheumatologic pathology (Figure 3B) [27,28,29].

### 2.3. Metabolic Diseases

The chemotactic effects of SRGN expand to various pathological conditions related to inflammation, including obesity. SRGN expression has been associated with an inflammatory transcription program in white adipose tissue in mice, linked to macrophage concentration and perturbed tissue integrity. Furthermore, SRGN silencing negatively impacted the size of adipocytes and, conversely, mature adipocytes downregulated SRGN expression in vivo. Bariatric surgery and weight loss attenuated SRGN-driven inflammation and manifested positive outcomes for patients experiencing obesity-related illness, highlighting the clinical relevance of SRGN [30]. In line with these findings, SRGN, PF4 and asporin PG have emerged as useful biomarkers for laparoscopic sleeve gastrectomy efficiency in cases of nonalcoholic fatty liver disease. Serum levels of SRGN and PF4 were found to be elevated in disease-associated patients, while low asporin levels predicted gastrectomy efficacy more accurately [31]. The detection and evaluation of serum markers have also pinpointed important insights into discrimination between metabolic irregularities, among others, diabetic-related retinopathy. Proliferative and non-proliferative diabetic retinopathy patients have been shown to possess high levels of serum SRGN compared with healthy controls and type-2 diabetes mellitus patients, providing a useful biomarker for differentiating the underlying metabolic profile and pathology leading to blood vessel and eye disease. In vitro experiments showcased the apparent promotion of SRGN expression upon high-glucose treatment, accompanied by apoptosis [32].

### 2.4. Cardiovascular Diseases

Given the inflammatory contribution to the atherosclerotic microenvironment, SRGN has also been studied for its potential implications in progression and prediction of myocardial pathologies. The SRGN-dependent granule organization and secretion have been shown to stabilize fibrin regardless of heart disease. In parallel, high SRGN has been positively correlated with high troponin and CRP levels in the serum of patients with ST-segment elevation myocardial infarction compared with control samples [33]. Secreted SRGN was also found to be higher in asymptomatic patients’ atheromatic plaques compared with symptomatic patients, acquiring HS/CS glycanation and accumulating together with macrophages and oxidized lipids. The inflammatory cellular components of the atherosclerotic ECM seem to produce and secrete SRGN to regulate inflammatory mediators’ bioavailability, among others ILs, cytokines and TGF-β, which were also found to be correlated with SRGN levels in atherosclerotic plaques [34]. Different studies have also demonstrated that high SRGN levels are associated with upregulated IL-6/8 and TNF-α in DNA methyltransferase 3A (DNMT3A) mutant monocytes, initiating inflammatory paracrine signaling and exacerbating overall heart failure disease and genetic predisposition [35,36].

### 2.5. Neuroinflammation and Neurodegenerative Diseases

Proteomics analysis has emerged as a valuable tool to evaluate the prognostic value of biomarkers in the battle against neurodegenerative disease. Regarding Alzheimer’s disease, cerebrospinal fluid and serum provide selective and achievable sources of information respectively, highlighting the underlying pathology. In detail, patients with Alzheimer’s disease alter their N-glycan protein synthesis pathways, exhibiting perturbed ATP synthesis, inflammatory and antioxidant response, reduced SRGN levels in their cerebrospinal fluid and significantly elevated levels in their serum. Although there has not been a rationale determining the impact of SRGN on this condition, it is well defined that HS-PGs participate in amyloid fibril formation in Alzheimer’s disease, and SRGN has been detected as overexpressed throughout all tissue lesions [37,38]. Moreover, the inflammatory ECM constitutes a main driver in intracranial aneurysm and potentiates its severity, as indicated by the apparent upregulation of SRGN, CXCL-16 and fibronectin secreted by monocytes and macrophages, compared with normal surrounding cells [39]. Microglia cells also stimulate neuroinflammatory cues post-ischemic stroke, accompanied by SRGN and CD44 overexpression. Lipopolysaccharide treatment has been shown to induce SRGN expression, and, hence, SRGN/CD44/NF-kB signaling and HIF-1-dependent glycolysis, leading to suppressed injury repair in vitro and in vivo, highlighting, in total, the therapeutic perspectives of targeting extracellular SRGN (Figure 3C) [40]. Accordingly, the SRGN/CD44 axis has been found to stimulate astrocytes’ proliferation capacity and mimic epithelial–mesenchymal transition (EMT), inhibiting their maturation in spinal cord injury. This transcriptional and phenotypic reprogramming was found to be SRGN/collagen-dependent, as well as SRGN/CD44/Akt-mediated, to restore the glial niche and augment tissue repair (Figure 3C) [41].

## 3. SRGN Coordinates Inflammation and Oncogenic Signaling in Cancer

The tumor microenvironment (TME) displays a pivotal role in cancer progression by promoting cell signaling and ECM remodeling, associated with the aggressiveness of malignant cells. SRGN is a key player in malignancy, synchronizing pro-tumorigenic inflammatory responses, as well as inflammatory-independent cascades [9]. CS-PGs, namely, SRGN, biglycan and decorin, have presented significant induction in GBM tissue specimens compared with healthy brain tissue, enhancing the already existing glycan ECM content [42]. Similarly, SRGN expression has been found to be significantly elevated in the serum of cancer patients, such as ovarian cancer, acute myeloid and lymphoblastic leukemia, breast cancer and hepatocellular carcinoma [43,44,45]. In ovarian cancer and non-malignant endometriosis, SRGN was upregulated together with decorin and glypican-3/5 PGs, accompanied by cancer-associated stimulation of hypoxia, glycolysis and Wnt-related markers, highlighting the probable implication of PGs in the overall cancer cell phenotype [4,46,47].

### 3.1. Regulation of Immune Response

SRGN expression is consistently induced in cell populations in malignant tissues. Macrophages present in the TME of liver tissue secrete high levels of SRGN throughout their entire cell cycle compared with healthy controls, while cancer HepG2 hepatocellular cells stimulate vascular endothelial GF (VEGF) signaling, angiogenesis and drug resistance upon SRGN secretion in vitro and in vivo. This autocrine function is simultaneous with paracrine signaling of SRGN to tumor-associated macrophages (TAMs), resulting in STAT3/NF-kB activation and macrophage polarization to promote HepG2 proliferation, migration and invasion (Figure 3D). It is also highly probable that TAMs are initially recruited due to enhanced SRGN secretion from cancer cells in their TME [48]. SRGN-driven immune evasion of tumor cells has also been described in gastric cancer via enrichment of the TME with CD4+ regulatory T cells (Tregs). Tregs overexpress the CD44 receptor and transduce SRGN signaling through TGFBRI/Smad3 for Treg differentiation, reactive oxygen species (ROS) neutralization, and glycolysis. This axis was activated only in lymphocyte activation gene 3-positive (LAG3+) Treg cells, while limited oxidative stress through SRGN further enhanced LAG3 expression, forming an oncogenic SRGN/LAG3 positive feedback loop [49]. In accordance with the above, high SRGN expression is representative of a malignant phenotype in cases of metastatic skin cutaneous melanoma and has been shown to organize immune cells’ infiltration within the TME [50].

### 3.2. Cell Signaling, EMT and Stemness

SRGN expression has also been negatively correlated with thyroid transcription factor-1 (TTF-1) levels in lung adenocarcinoma, a combination predicting poor patient survival [51]. SRGN function induces programmed death ligand-1 (PDL-1), IL-6/8 and CXCL-1 expression towards immune evasion, proliferation and migration of both cancer cells and cancer-associated fibroblasts (CAFs). SRGN induction was also described to be downstream of TTF-1 regulation in TTF-1-negative populations, and, more precisely, being dependent upon perturbed methionine metabolism, abolition of *SRGN* promoter methylation, and nuclear factor erythroid 2-related factor 2 (Nrf-2) TF activity (Figure 3D) [52]. The interplay between SRGN/inflammation/metabolism has also been studied in hepatocellular carcinoma, suggesting that SRGN promotes inflammatory response, CD44 expression and mitogen-activated protein kinase (MAPK) signaling, as well as EMT through E-/N-cadherin, vimentin and MMP-2 regulation to promote migration and metastasis in vitro and in vivo. The mesenchymal properties of cancer cells were maintained by transcriptional coactivator YES-associated protein (YAP) activation through gene expression promotion, dephosphorylation and nuclear translocation towards TF activity in SRGN-expressing cells. YAP activation induced the expression of cysteine-rich secretory protein LCCL domain-containing 2 (CRISPLD2), a downstream effector of CD44 related to hepatocellular cancer aggressiveness. Altogether, SRGN/CD44/YAP/CRISPLD2 interplay concluded in tumor progression and simultaneous metabolic reprogramming, with SRGN enhancing the potential of all the above mediators (Figure 3D) [53].

The correlation between SRGN levels and breast cancer aggressiveness has led to further investigation of its pleiotropic roles. Triple-negative breast cancer (TNBC) cells depend on SRGN expression to retain their stemness capacity by modulating EMT factors, TGF-β signaling, and shaping overall the predispositions for their metastatic potential. Infiltrating immune cells in breast tumor tissue are another important source of serglycin in the TME that support cancer cells’ potential [54]. SRGN function also maintains high levels of IL-8 in the TNBC ECM, together with its receptor CXCR-2, thus stimulating downstream signaling cascades, including PI3K, Src and Rac signaling for cancer progression and drug resistance (Figure 3D) [55]. A similar trend has been observed in glioblastoma (GBM) cells, expressing high levels of SRGN, ILs and their receptors and matrix-degrading enzymes, displaying profound proliferation, migration, invasion and stemness capacity. SRGN depletion in GBM cells corresponded to differentiation towards the epithelial-like astrocytic phenotype, significant attenuation of cell aggressiveness, reduced MAPK/Src/STAT3 signaling and downregulation of key inflammatory mediators in vitro and in vivo (Figure 3D) [56].

It is evident that SRGN displays its function at multiple levels of action, with CD44 possessing a central role in mediating extracellular signals. SRGN directly interacts with secreted midkine protein through its CS chains to stabilize SRGN/midkine/CD44 coupling. This results in esophageal cancer progression in vitro and in vivo, through MAPK signaling, concomitant c-Myc stabilization, MMPs’ secretion and SRGN-dependent activation [57]. Among others, SRGN has also been correlated with epidermal growth factor receptor (EGFR) and Ki67 protein to maintain cell viability of TNBC cells and prevent anoikis. Alongside these, the stemness potential of breast cancer cells heavily depends on SRGN/CD44/MAPK/β-catenin signaling (Figure 3D) [58]. CS/HS-SRGN secreted within the non-small-cell lung cancer TME interacts with CD44 to stimulate Src and FAK transactivation, therefore uncoupling paxillin/FAK and substantiating cell migration. FAK/Rho-induced cytoskeleton remodeling was also accompanied by lamellipodia and filopodia formation to further enhance cell motility (Figure 3D) [59]. In bone marrow malignancies, such as myeloid leukemia, CD45 correlates with SRGN and CD44 expression and drug resistance, suggesting potential interplay between cell-specific markers and established SRGN/receptor interactions in disease progression [60].

Cathepsin L (CTSL) has emerged as a new SRGN regulator, promoting SRGN expression and secretion in neuroblastoma cells. This induction was parallel with the drug resistance-associated transcription program, together with autophagic cell death and apoptosis inhibition. CTSL/SRGN cooperation led to cisplatin/doxorubicin resistance, tumor growth and predicted poor survival in patients [61]. Apoptosis suppression through SRGN function has also been observed in osteosarcoma tumors, overexpressing SRGN and upregulating the JAK/STAT pathway, together with anti-apoptotic B-cell lymphoma-2 (Bcl-2) marker, compared with normal osteocytes [62]. Extracellular SRGN in breast cancer cells can also trigger integrin α5 (ITGA5) outside-in signaling, mediated by FAK and cAMP response element-binding protein (CREB), leading to YAP upregulation. In turn, YAP synergizes with TEA domain family member 1 (TEAD1) to stimulate SRGN expression by binding to the *SRGN* promoter, while YAP/RUNX1-dependent TF activity enhances drug resistance. SRGN/YAP/TEAD1/RUNX1 interplay highlights the upstream effect of SRGN via integrins to modulate distinct TF activity, predominantly YAP, also forming a positive feedback loop. Eventually, this SRGN-induced axis promoted drug resistance and maintained the oncogenic and mesenchymal properties of breast cancer cells. Interestingly, SRGN directly interacted with ITGA5, acting as a ligand in a CD44-independent manner, raising further inquiries regarding possible novel SRGN receptors, as suggested by independent studies providing evidence for direct SRGN/ITGB1 interaction (Figure 3D) [9,63].

## 4. SRGN Potentiates Cancer–Stromal Cell Communication

CAFs’ stimulation has been implicated in various aspects of tumor progression by executing signal transduction initiated by cancer cells and vice versa. In tissue samples of patients with oral squamous-cell carcinoma, CAF subtypes have been shown to differentially express and secrete SRGN under both normoxia and hypoxia. Interestingly, hypoxia induces a non-canonical secretory pathway, in which SRGN secretion depends on autophagic flux but not lysosomal recycling or endoplasmic reticulum translocation. Extracellular SRGN then interacts with MMP-2/9 and potentiates ECM degradation and cell migration [64,65]. The autophagic secretion occurred only in CAFs, co-expressing SRGN and microtubule-associated protein 1A/1B-light chain 3 (LC3), whereas cancer cells exhibited resistance to autophagy induction. Autophagy inhibition highlighted that SRGN secretion is a downstream effect of autophagy-related 5 (ATG5) and beclin-1-dependent autophagic switch, leading to SRGN localization to the Golgi apparatus and autophagic secretion under hypoxic conditions [65].

In different cases, such as head and neck squamous carcinoma, both cancer cells and CAFs secrete excessive SRGN under the guidance of HIF-1 function in hypoxia. Stromal cells’ contribution pinpointed that TME SRGN encourages tumor growth through paracrine activation of Wnt/β-catenin signaling in cancer cells (Figure 3D) [66]. Different studies have supported the proposition that HIF-1 directly binds to the *SRGN* promoter in colorectal cancer cells to induce gene transcription and aggressiveness [67]. SRGN levels were also correlated with stimulated TGFBRI-associated signaling that drives proliferation, invasion, proteolytic and inflammatory potential in GBM cells. SRGN also promotes fibroblasts’ activation in a paracrine fashion and results in SRGN-dependent induction of inflammatory mediators and MMPs. The activation of fibroblasts by GBM cells also depends on active CXCR-2 signaling both in GBM cells and fibroblasts (Figure 3D) [68]. The inflammatory response driven by SRGN in esophageal cancer cells has been shown to provoke IL-1β expression and secretion, followed by CAFs stimulation and aberrant secretion of hepatocyte growth factor, enhancing the overall aggressiveness and angiogenetic properties of cancer cells [24]. In parallel, gastric cancer cells and patient tissues exhibit high levels of extracellular SRGN binding to CAFs’ CD44. This interaction induces successive transcriptional upregulation of c-Myc/lysine demethylase 5B (KDM5B)/IL-8 expression in SRGN-treated Hs738 fibroblast cells, associated with poor prognosis in gastric cancer [69].

Collaterally with the above, SRGN overexpression in astrocytes has been involved in GBM tumor growth initiated by astrocytic stimulation in the TME [70]. In giant cell tumor of bone, neoplastic stromal cells provoke multinucleated giant cells’ oncogenic transformation and recruit secreted SRGN for targeted differentiation of monocytes into osteoclasts in vitro and in vivo. This process accelerates tumor progression and bone degeneration upon SRGN binding to monocytes’ CD44 and focal adhesion kinase (FAK) activation [71]. Lastly, SRGN, cation-dependent mannose-6 phosphate receptor (M6PR) and ephrin type-B receptor 4 (EphB4) markers have been found elevated in the serum of patients with esophageal squamous-cell carcinoma, predicting poor overall survival. This correlation has been shown to implicate SRGN as a regulator of cancer cell exosomes’ cargo, being enriched with both M6PR and EphB4 to promote cell survival, migration and invasion (Figure 3D) [72].

## 5. Epigenetic Regulation of SRGN

Recent studies have underlined the implication of miRNAs in SRGN regulation in malignancies. In acute myeloid leukemia patients, high SRGN levels were correlated with elevated small nucleolar RNA host gene 3 (SNHG3), a long non-coding RNA associated with poor prognosis. SNHG3 overexpression was found to directly neutralize onco-suppressive miR-758-3p, known to bind and inhibit *SRGN* mRNA translation. In this context, simultaneous SRGN/SNHG3 function inhibited miR-758-3p and amplified the oncogenic potential in vitro and in vivo, while also suppressing caspase-dependent apoptosis [73]. miR-26b-5p has been characterized as a suppressor of SRGN expression through binding to the 3′-untranslated region of *SRGN* mRNA, leading to attenuated aggressiveness of drug-resistant breast cancer cells in vitro and in vivo. In breast cancer patients, SRGN prevails upon the inhibitory miR-26b-5p function, while the relevant underlying mechanism still remains unknown [74]. Similarly, in nasopharyngeal carcinoma, SRGN and CREB1 were found to be overexpressed, and miR-148a-5p was found to be decreased. Mechanistically, miR-148a-5p recognizes *CREB1* mRNA, thus inhibiting its expression, whereas CREB1 induces SRGN transcription by binding to its promoter. In normal hepatocytes, low basal levels of active STAT result in forkhead box protein O1 (FoxO1) interaction with the miR-148a-5p promoter to amplify its transcription, thereby downregulating CREB1/SRGN levels. In nasopharyngeal carcinoma cells, SRGN dispossesses FoxO1/miR-148a-5p onco-suppressive roles and stimulates proliferation, migration and invasion in vitro and in vivo [75].

## 6. Conclusions

Cell–ECM interplay provides a potent microenvironment for cellular responses and tissue fidelity, with SRGN emerging as a significant factor in disease progression. High levels of SRGN expression and secretion, as well as high levels detected in biological fluids, serve as useful tools for evaluating disease stage and pathology, pinpointing its diagnostic and predictive importance in inflammatory-related and malignant diseases. In accordance with the above, the active cascades initiated by extracellular SRGN highlight future therapeutic strategies for SRGN neutralization and cell-signaling disruption through function-blocking monoclonal antibodies and gene editing. Interestingly, it has become increasingly evident that SRGN tethers to distinct receptors apart from CD44, among others, integrin subunits, suggesting diverse transmembrane moieties involved in SRGN signaling. Considering the plethora of SRGN binding partners characterized, it is imperative to explore how these interactions are accompanied by the formation of local signaling networks and concomitant intracellular ramifications. The predominant GAG-dependent function of SRGN, alongside its protein’s core importance in MMPs’ regulation, indicates new undisclosed roles regarding the duality in SRGN structure and function. This duality also expands to the simultaneous presence of SRGN within the ECM and intracellularly, underscoring the broad range of phenomena in which it participates. Furthermore, the limited but crucial knowledge concerning the correlation between SRGN and exosomes’ cargo advocates for the investigation of its roles in vesicle packaging and paracrine communication. The abundance of miRNAs in extracellular vesicles and the dominance of SRGN in inhibiting onco-suppressive epigenetic regulation of cancer cells could also propose a cooperative relationship between the two. As proposed in different studies, SRGN coincides with an inflammatory phenotype and alterations in autophagic flux in a context-dependent manner, while its implication in the secretory pathway could imply new insights into protein recycling, turnover and proteostasis.

## Data Availability

No new data were created or analyzed in this study. Data sharing is not applicable to this article.

## Abbreviations

ADAMTS14	A Disintegrin and Metalloproteinase with Thrombospondin Motifs 14
ATG5	Autophagy-Related 5
ATP	Adenosine Triphosphate
Bcl-2	B-Cell Lymphoma 2
CAFs	Cancer-Associated Fibroblasts
CCL3	C-C Motif Chemokine Ligand 3
CCL26	C-C Motif Chemokine Ligand 26
C/EBPα	CCAAT/Enhancer-Binding Protein Alpha
CREB	cAMP Response Element-Binding Protein
CREB1	cAMP Response Element-Binding Protein 1
CRISPLD2	Cysteine-Rich Secretory Protein LCCL Domain-Containing 2
CRP	C-Reactive Protein
CS	Chondroitin Sulfate
CTSL	Cathepsin L
CXCL-2	C-X-C Motif Chemokine Ligand 2
CXCL-16	C-X-C Motif Chemokine Ligand 16
CXCR-2	C-X-C Chemokine Receptor 2
DNMT3A	DNA Methyltransferase 3A
ECM	Extracellular Matrix
EGFR	Epidermal Growth Factor Receptor
EMT	Epithelial–Mesenchymal Transition
EphB4	Ephrin Type-B Receptor 4
EVs	Extracellular Vesicles
FAK	Focal Adhesion Kinase
FDR	False Discovery Rate
FoxO1	Forkhead Box Protein O1
GAGs	Glycosaminoglycans
GBM	Glioblastoma
GFs	Growth Factors
Hep	Heparin
HIF-1	Hypoxia-Inducible Factor 1
HS	Heparan Sulfate
HS-PGs	Heparan Sulfate Proteoglycans
HYALs	Hyaluronidases
IL-1β	Interleukin-1 Beta
IL-6	Interleukin-6
ILs	Interleukins
ITGA5	Integrin Alpha 5
ITGB1	Integrin Beta 1
JAK/STAT	Janus Kinase/Signal Transducer and Activator of Transcription
LAG3	Lymphocyte Activation Gene 3
LC3	Microtubule-Associated Protein 1A/1B-Light Chain 3
MAPK	Mitogen-Activated Protein Kinase
M6PR	Mannose-6-Phosphate Receptor
MCs	Mast Cells
miRNAs	MicroRNAs
MKs	Megakaryocytes
MMPs	Matrix Metalloproteinases
NF-κB/NF-kB	Nuclear Factor Kappa B
Nrf-2	Nuclear Factor Erythroid 2-Related Factor 2
OA	Osteoarthritis
PDL-1	Programmed Death Ligand-1
PF4	Platelet Factor 4
PGs	Proteoglycans
PI3K	Phosphoinositide 3-Kinase
Rac	Ras-Related C3 Botulinum Toxin Substrate
ROS	Reactive Oxygen Species
RUNX1	Runt-Related Transcription Factor 1
SNHG3	Small Nucleolar RNA Host Gene 3
Src	Proto-Oncogene Tyrosine-Protein Kinase Src
SRGN	Serglycin
STAT3	Signal Transducer and Activator of Transcription 3
TAMs	Tumor-Associated Macrophages
TEAD1	TEA Domain Family Member 1
TF	Transcription Factor
TGF-β	Transforming Growth Factor-Beta
TME	Tumor Microenvironment
TNBC	Triple-Negative Breast Cancer
TNF-α	Tumor Necrosis Factor-Alpha
Tregs	Regulatory T Cells
TTF-1	Thyroid Transcription Factor-1
VEGF	Vascular Endothelial Growth Factor
YAP	YES-Associated Protein
